# Temporal influence of endocrine therapy with tamoxifen and chemotherapy on nutritional risk and obesity in breast cancer patients

**DOI:** 10.1186/s12885-017-3559-z

**Published:** 2017-08-29

**Authors:** Mariana Tavares Miranda Lima, Kamila Pires de Carvalho, Fernanda Silva Mazzutti, Marcelo de Almeida Maia, Paula Philbert Lajolo Canto, Carlos Eduardo Paiva, Yara Cristina de Paiva Maia

**Affiliations:** 1 Graduate Program in Health Sciences, Federal University of Uberlandia, Avenida Pará, 1720 Bloco 2U, Campus Umuarama, Uberlandia, Minas Gerais CEP 38400-902 Brazil; 2 Nutrition Course, Medical Faculty, Federal University of Uberlandia, Avenida Pará, 1720 Bloco 2U, Campus Umuarama, Uberlandia, Minas Gerais CEP 38400-902 Brazil; 3 Faculty of Computing, Federal University of Uberlandia, Avenida Joao Naves de Avila, 2121, Campus Santa Monica, Uberlandia, Minas Gerais CEP 38400-902 Brazil; 4 Department of Clinical Oncology, Clinic’s Hospital, Federal University of Uberlandia, Avenida Pará, 1720, Setor de oncologia, sala 9 Campus Umuarama, Uberlandia, Minas Gerais CEP 38.405-320 Brazil; 5 Department of Clinical Oncology, Graduate Program in Oncology, Palliative Care and Quality of Life Research Group (GPQual), Pio XII Foundation - Barretos Cancer Hospital, Rua Antenor Duarte Vilela, de 1301/1302 ao fim, Doutor Paulo Prata, Barretos, Sao Paulo CEP 14784-400 Brazil

**Keywords:** Breast neoplasm, Endocrine therapy, Tamoxifen, Chemotherapy, Body composition, Body weight

## Abstract

**Background:**

The effect of endocrine therapy with tamoxifen (TMX) on weight gain has been reported in the literature, but the outcomes are still controversial. Moreover, previous treatment options, such as chemotherapy (CT), also include body changes. The focus of this study was to verify the temporal influence of endocrine therapy with TMX on nutritional risk and obesity and its association with CT in breast cancer patients.

**Methods:**

In this cross-sectional study, 84 breast cancer surviving women were evaluated during endocrine therapy with TMX. Anthropometric, biochemical and body composition parameters were measured. A generalized estimating equation (GEE) was used to examine the association between CT and groups of women using TMX categorized by the duration of the treatment (group 1, women using TMX for the first 3 years; group 2, women using TMX between 3 and 4 years and group 3, women using TMX for more than 4 years).

**Results:**

The interaction of CT with duration of TMX use showed a significant effect on Body Mass Index (BMI), waist circumference (WC) and body fat percentage (BFP) (GEE *p*-value = 0.002, 0.000, 0.000, respectively). Women from group 1 who underwent CT presented higher values of body variables compared to those women from group 2 who also underwent CT (BMI = 29.14 ± 0.93, 26.76 ± 0.85 kg/m^2^; WC = 94.45 ± 1.96, 91.07 ± 2.44 cm; BFP = 36.36 ± 1.50, 33.43 ± 1.66%, respectively). On the other hand, women from group 1 who did not undergo CT presented lower values of body variables compared to those women from group 2 who also did not undergo CT (BMI = 25.29 ± 0.46, 28.40 ± 0.95 kg/m^2^; WC = 85.84 ± 0.90, 97.75 ± 0.88 cm; BFP = 30.32 ± 0.43; 42.95 ± 1.03%, respectively).

**Conclusions:**

Women on endocrine therapy with TMX are mostly overweighed and obese, most evidently in women who received CT, and who were at the beginning of treatment. Women that did not undergo CT, despite presenting lower values of body variables in the first 3 years, still deserve special attention because significantly higher values were observed in women between 3 and 4 years of therapy.

## Background

Breast cancer (BC) accounts for 29% of all new cases of cancer in women, being the second leading cause of death [1]. In patients treated with surgery, adjuvant endocrine therapy with tamoxifen (TMX), a selective estrogen receptor modulator, has been widely used in individuals expressing estrogen and/or progesterone endocrine receptors [2], prolonging substantially disease-free intervals and survival outcomes [3].

Changes in body weight are described as side effects during treatment [4–6]. Both the initial overweight and the amount of weight gained during treatment negatively influence the prognosis, survival and quality of life of women with BC [7–9]. In endocrine therapy, even though this gain is more modest (1 to 2 kg) [10, 11] when compared to the CT period (3 to 7 kg) [12–14], it is a major concern regarding non-adherence to endocrine therapy [15]. Furthermore, even without weight gain, these women are affected by changes in body composition with loss of muscle mass and an increase in body fat percentage (BFP) [10, 16]. The excess of BFP in postmenopausal women results in increased estrogen and androgen concentrations in adipose tissue [17], which can stimulate cancer cells [18], change circulating levels of pro-inflammatory cytokines [19], and also impact the efficiency of TMX [20]. However, these results are still unclear and need to be further investigated.

Furthermore, metabolic implications at the beginning of treatment for BC reveal impairment of glucose metabolism and dyslipidemia [21] and extend into survivors on endocrine therapy with TMX [22–24]. These implications are important along with weight gain due to the occurrence of cardiovascular diseases that may develop over time in postmenopausal women on endocrine therapy with TMX [25, 26]. However, even in face of these implications, the overall beneficial effects of treatments for BC are already established [2, 3]. Also, the combination of treatments for BC, such as chemotherapy (CT) plus TMX, promotes substantial benefits compared to CT alone, producing a further reduction in recurrence risk [2].

Considering the recommendation to use endocrine therapy with TMX for up to 10 years [3], the impact of body modifications on survival and disease recurrence during endocrine therapy is poorly understood [27, 28]. In this sense, knowing the potential long-term effect of previous treatments, such as CT [12, 13], it is necessary to understand its influence on the TMX side effects related to anthropometric parameters and BFP at different moments of endocrine therapy. In addition, this understanding will enable the development of multidisciplinary interventions directed throughout the treatment.

We hypothesized that women who underwent CT were more obese and that the degree of obesity was more evident at the beginning of TMX therapy. Thus, the objective of this study was to analyze the temporal influence of endocrine therapy with TMX on nutritional risk and obesity and its association with CT in BC patients, evaluated by means of anthropometric variables and body composition.

## Methods

### Ethical aspects

A transversal study conducted in 2015–2016 in a brazilian university hospital (HC-UFU, Uberlandia, Minas Gerais, Brazil) including one assessment with BC patients during endocrine therapy with TMX, in the period from August 2015 to March 2016.

This study was approved by the Human Research Ethics Committee (protocol number 907.129/14) and the entire study was conducted based on the standards of the Helsinki Declaration. All participants signed a free and informed consent form.

### Sample size calculation

The sample size required for this study was determined using the G*Power software, version 3.1 [29]. The sample size calculations were based on an F test linear multiple regression with effect size f of 0.15, an alpha level of 0.05, 95% power and 3 predictors. Given the output Parameter, a total sample of 84 women was required at final analysis.

### Eligibility criteria

The study included women diagnosed with BC with indication of endocrine therapy with TMX and with verbal and cognitive capacity to respond to the instruments used for data collection. Women older than or equal to 80 years and less than or equal to 18 years were excluded from the study, as well as patients with locoregional or distant BC recurrence; diagnosis of any other type of cancer; autoimmune diseases and/or use of corticosteroids; presence of diabetes mellitus; thyroid diseases; depressive syndrome; pregnant or postpartum women; admission to palliative care programs; institutionalized patients; without telephone contact; previous use of TMX and/or change to the use of aromatase inhibitors.

### Participants for recruitment

**Fig. 1:**
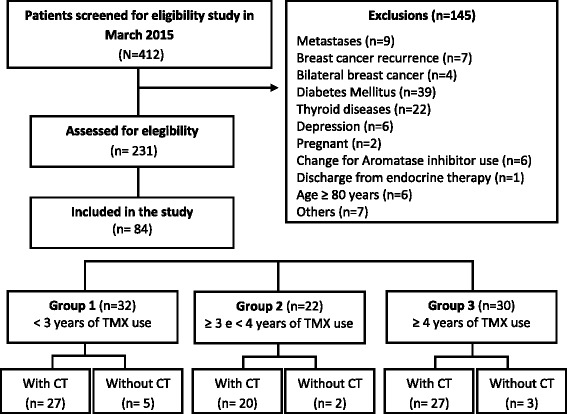
Diagram reporting the number of women screened and recruited in this study (*n* = 84). Diagram reporting the number of women with breast cancer on endocrine therapy with tamoxifen screened and recruited during the study conducted at a university hospital in the city of Uberlandia, Minas Gerais, Brazil, 2015–2016 (*n* = 84). Group 1, women using tamoxifen for the first 3 years; group 2, women using tamoxifen between 3 and 4 years; Group 3, women using tamoxifen for more than 4 years; CT, chemotherapy; TMX, tamoxifen

The active medical records of patients being treated with TMX in the month of March 2015 were analyzed (*n* = 412) and 231 patients were classified as eligible for the study. Using a table of random numbers, 84 patients were invited to participate in the study according to the previously calculated sample. Groups were set according to the duration of TMX use, obtained by stratification into tertiles at three times of use (groups 1, 2 and 3), considering equivalent ranges of the duration: group 1 included 32 women using TMX for the first 3 years; group 2 included 22 women using TMX between 3 to 4 years; and group 3 included 30 women using TMX for more than 4 years (maximum time equals to 6 years and 6 months). The three groups included, after strict eligibility criteria, both women who underwent chemotherapy along with those who did not undergo (Fig. 1). The invitation to participate was made by phone and the evaluations were carried out at the oncology department of the clinical hospital.

### Anthropometric assessment

A mechanical scale was used to measure weight, with sensitivity of 100 g; for height, a vertical stadiometer with a 1 mm precision scale was used; and for waist circumference (WC) a flexible and inelastic tape was used, following the protocol recommended by the World Health Organization [30]. After obtaining these measurements, the Body Mass Index (BMI) were calculated dividing weight by height squared (Kg/m^2^), taking into consideration elderly women over 60 years of age [31].

The horizontal tetra polar bioelectrical impedance (BIA) (Biodynamics device model 450) was used to evaluate body compartments, using the cutoff point for excess BFP in women ≥ 24% [32]. Participants were guided regarding the protocol of the test [33].

### Quantitative dietary assessment

Properly trained nutritionists collected information about food consumption by means of a 24-h dietary recall (24HR) applied through telephone interviews, according to the technique used in the Vigitel Study [34] with adaptations. For each participant, three nonconsecutive 24HR were applied, including a day of the weekend, in order to better reflect the eating habits of the participants. From the 24HR, the mean quantity of total energy, carbohydrate, protein and lipid were estimated. Quantification of nutrients was performed through Dietpro® software, version 5.7, using as a reference, preferably, the Brazilian Table of Food Composition [35]. However, for those foods not found in this table, the international reference was used, the table from the United States Department of Agriculture [36].

### Laboratory assays

Venous blood was collected at the time of the interview, between 7 am and 10 am, after overnight fasting and under standard conditions for analysis of Total Cholesterol, LDL Cholesterol (LDL-C), HDL Cholesterol (HDL-C) (mg/dL), TG (mg/dL), Fasting glucose (mg/dL), C Reactive Protein (CRP) (mg/dL), and a complete blood count. The results were evaluated according to recommendations established in the literature [37–39].

### Statistical analyses

First, the Kolmogorov-Smirnov normality test was performed. Parametric tests for variables with normal distribution, or non-parametric tests for variables without normal distribution were performed. Generalized Estimating Equations (GEE) were used to examine the association between groups of TMX/CT and nutritional risk and obesity at first, second and third usage time adjusting for age, smoking, alcohol consumption, physical activity, energy (kcal), and clinical stage. An interaction term between the CT and time was included in the model. The GEE model accounts for correlations among the within-subject outcome variables of BMI, WC and BFP and provides consistent estimates of the parameters of the standard errors using robust estimators. The adjustment method for multiple comparisons was Sequential Sidak. All statistical analyses were run using the SPSS® (SPSS, Inc., Chicago, USA) software package (SPSS Statistics for Windows, version 21) and a *p*-value ≤0.05 was considered statistically significant.

## Results

The study included 84 women with mean age of 53.11 ± 8.73 years. Socio-demographic, clinical, hormonal and therapeutic characteristics are presented in Table 1. Most women (52.4%, *n* = 44) considered themselves white, reported monthly income higher than 3 minimum wages (46.5%, *n* = 39) and low education level (42.9%, *n* = 36). Regarding clinical and hormonal characteristics, 91.7% (*n* = 77) were found to be postmenopausal and 90.5% (*n* = 76) presented invasive ductal carcinoma. As for the molecular phenotype, the majority (51.2%, *n* = 43) was classified as luminal B. Regarding surgical procedures, 52.4% (*n* = 44) of the women underwent conservative breast surgery and 46.5% (*n* = 39) had mastectomy. The percentage of patients submitted to adjuvant chemotherapy was 58.3% (*n* = 49), 29.8% (*n* = 25) to the neoadjuvant and 11.9% (*n* = 10) did not undergo chemotherapy. The majority were treated with adriamycin + cyclophosphamide + docetaxel (AC-T) regimen (42.9%, *n* = 36) followed by cyclophosphamide, doxorubicin and 5-fluorouracil (FAC) (25.0%, *n* = 21).

**Table 1:** Sociodemographic, clinical, hormonal and therapeutic characteristics (*n* = 84)

Characteristics	n (%)
Race	
White	44 (52.4)
Black	9 (10.7)
Hispanic	31 (36.9)
Income, R$^a^	
440–880	14 (16.7)
881–1.760	31 (36.9)
> 1.761	39 (46.5)
Education	
Elementary School - Incomplete	36 (42.9)
Elementary School - Complete	9 (10.7)
High School - Incomplete	6 (7.1)
High School - Complete	21 (25.0)
Graduate degree	12 (14.3)
Menopausal status	
Premenopausal	7 (8.3)
Postmenopausal	77 (91.7)
Tumoral Subtype	
Ductal	76 (90.5)
Lobular	4 (4.8)
Mucinous	3 (3.6)
Ducto-Lobular	1 (1.2)
Clinical Stage	
I	21 (25.0)
II	49 (58.3)
III	14 (16.7)
Tumor grade	
G1	11 (13.1)
G2	61 (72.6)
G3	7 (8.3)
NR	5 (6.0)
Molecular Subtypes	
Luminal A	37 (44.0)
Luminal B	43 (51.2)
NR	4 (4.8)
Surgery	
Breast-conserving surgery	44 (52.4)
Mastectomy	39 (46.5)
No surgery	1 (1.2)
Chemotherapy	
Adjuvant	49 (58.3)
Neoadjuvant	25 (29.8)
No chemotherapy	10 (11.9)
Chemotherapy Regimen	
AC + Docetaxel	36 (42.9)
FAC	21 (25.0)
CMF	18 (21.4)

*NR* not reported, *G1* well-differentiated tumor (low grade), *G2* moderately differentiated tumor (intermediate grade), *G3* poorly differentiated tumor (high grade), *AC* adriamycin + cyclophosphamide, *FAC* cyclophosphamide, doxorubicin, and 5-fluorouracil, *CMF* cyclophosphamide, methotrexate, and 5-fluorouracil

^a^Minimum wage per month, R$ 880,00

Regarding the anthropometric parameters, the current BMI values 63.1% of participants were above the values of eutrophy for adults and elderly (26.79 ± 4.59; 28.16 ± 4.53 kg/m^2^, respectively). When comparing the groups, the BMI values of adults were significantly higher among women in group 1 (28.38 ± 4.12 kg/m^2^, *p* = 0.018) when compared with the others. No statistically significant difference was found between the groups for the BMI of the elderly. In addition, among the BMI classifications, women who underwent CT (*n* = 74), 62.2% (*n* = 46) were classified as overweighed or obese and 37.8% (*n* = 28) were neither overweighed nor obese, considering adults and elderly. For those who did not undergo CT (*n* = 10), 70.0% (*n* = 7) were classified as overweight and 30.0% (*n* = 3) as non-overweighed. The BFP and WC presented mean values above the recommendations (35.23 ± 7.55%, 90.63 ± 11.07 cm, respectively), but without significant differences when compared between groups (Table 2).

**Table 2:** Characterization of the anthropometric and biochemical variables evaluated according to the groups established by the duration of tamoxifen use (*n* = 84)

Variables		Mean ± SD				
		Total (*n* = 84)	Group 1 (*n* = 32)	Group 2 (*n* = 22)	Group 3 (*n* = 30)	*p*-value
Age (range: 33–73 years)		53.11 ± 8.73	51.37 ± 7.35	54.09 ± 9.18	54.23 ± 9.69	0.37
Anthropometric						
Current BMI (Kg/m^2^)						
Adults (*n* = 62)		26.79 ± 4.59	28.38 ± 4.12	26.76 ± 4.29	24.54 ± 4.73	0.018
Elderly (*n* = 22)		28.16 ± 4.53	29.40 ± 7.87	27.30 ± 2.07	28.07 ± 3.86	0.760
WC (cm)		90.63 ± 11.07	93.11 ± 10.07	91.67 ± 10.83	87.21 ± 11.73	0.096
BFP (*n* = 74)		35.23 ± 7.55	35.55 ± 7.53	34.38 ± 7.45	35.54 ± 7.92	0.845
Biochemicals	Recommendation					
TG	<150 mg/dL	153.49 ± 85.21	156.43 ± 86.51	181.45 ± 102.83	130.03 ± 62.97	0.095
HDL-C	>60 mg/dL	55.19 ± 17.92	53.34 ± 16.62	47.51 ± 19.75^1^	62.78 ± 15.29^1^	0.006
LDL-C	<100 mg/dL	96.80 ± 28.24	94.79 ± 25.18	99.06 ± 37.88	97.30 ± 23.61	0.858
Blood glucose	<100 mg/dL	82.17 ± 30.22	78.41 ± 35.90	86.00 ± 29.02	83.37 ± 24.47	0.644
Hemoglobin	12.0 a 15.5 g/dL	12.88 ± 1.69	12.63 ± 2.54	13.00 ± 0.80	13.05 ± 0.83	0.582
WBC	3.500 a 10.500 mil/mm	5916.55 ± 1737.62	5403.12 ± 1589.95	6232.27 ± 1508.85	6232.67 ± 1954.68	0.104
Platelets	150 a 450 mil/mm	194,142.9 ± 65,099.0	185,062.50 ± 73,578.98	198,772.73 ± 68,529.40	200,433.33 ± 52.848.31	0.608
CRP	<0.3 mg/dL	0.42 ± 0.64	0.49 ± 0.98	0.37 ± 0.31	0.37 ± 0.30	0.698
Food consumption						
Energy (kcal)		1591.63 ± 526.80	1742.62 ± 580.62^2^	1393.56 ± 449.09^2^	1575.83 ± 482.39	0.054
Energy (kJ)		6659.38 ± 2204.13	7291,12 ± 2429,31^2^	5830,66 ± 1878,99^2^	6593,27 ± 2018,32	0.054
Carbohydrate (g)		201.94 ± 71.98	212.01 ± 67.38	182.85 ± 63.47	205.19 ± 81.60	0.331
Protein (g)		65.09 ± 25.02	72.14 ± 28.63	56.66 ± 21.86	63.76 ± 21.42	0.076
Lipids (g)		58.37 ± 22.69	66.74 ± 25.93^3^	48.61 ± 18.14^3^	56.61 ± 19.06	0.012

*BMI* body mass index, *WC* waist circumference, *TG* triglycerides, *HDL-C* high density lipoprotein, *LDL-C* low density lipoprotein, *WBC* white blood cell count, *CRP* C Reactive Protein, *SD* standard deviation, *Group 1* women using tamoxifen for the first 3 years, *Group 2* women using tamoxifen between 3 and 4 years, *Group 3* women using TMX for more than 4 years. The cutoff points of the biochemicals parameters were evaluated according to recommendations [37–39]; *p* < 0.05 was considered significant, calculated by ANOVA

^1^ *p* = 0.006; ^2^ *p* = 0.049; ^3^ *p* = 0.010

The blood analysis for the lipid parameters showed discretely altered values of TG and HDL-C (153.49 ± 85.21; 55.19 ± 17.92 mg/dL, respectively). Comparing the groups, significantly worse values of HDL-C in group 2 were observed compared to groups 1 and 3 (47.51 ± 19.75; 53.34 ± 16.62; 62.78 ± 15.29 mg/dL, *p* = 0.006, respectively). The same was not observed for TG when comparing the groups. For hemoglobin, WBC, platelets and CRP the values were within the recommended values (Table 2).

Regarding food intake, we did not find a statistically significant difference for the average amount of energy, carbohydrate and protein ingested among the three groups. However, lipids had significantly higher mean values in group 1 than in groups 2 and 3 (66.74 ± 25.93, 48.61 ± 18.14, 56.61 ± 19.06 g, respectively, *p* = 0.012).

In the GEE analyses, we did not find significant isolated effects of CT on BMI, WC and BFP (*p* = 0.102, *p* = 0.084, *p* = 0.607, respectively). However, significant effects were observed when we evaluated the duration of TMX use (determined by the three groups) on WC and BFP (*p* = 0.003 and *p* = 0.001, respectively). Furthermore, the interaction between these two factors (CT and duration of TMX use) was significant for all anthropometric and body composition parameters (*p* < 0.05) (Table 3).

**Table 3:** Model effect tests of tamoxifen use duration groups and whether or not chemotherapy is performed

Variable	Effect	df	Wald Chi-square	*p-value
BMI	Duration of use	2	3.16	0.206
	CT	1	2.68	0.102
	Duration of use x CT	2	12.31	0.002
WC	Duration of use	2	11.88	0.003
	CT	1	2.99	0.084
	Duration of use x CT	2	22.27	0.000
BFP	Duration of use	2	14.29	0.001
	CT	1	0.27	0.607
	Duration of use x CT	2	37.95	0.000

BMI, Body Mass Index; WC, waist circumference; BFP, body fat percentage; CT, chemotherapy; General Estimated Equations (GEE). Data adjusted for age, smoking, alcohol consumption, physical activity, energy (kcal), and clinical stage. df, Degree of freedom

*p values calculated by ANOVA

Table 4 shows the post hoc comparisons of the variables evaluated with CT and not CT and groups 1, 2 and 3. Analyses of the univariate effects showed that in group 1, women who did CT when compared with those who did not undergo CT, presented significantly higher values of BMI (29.14 ± 0.93; 25.29 ± 0.46 kg/m^2^, *p* = 0.003, respectively), WC (94.45 ± 1.96; 85.84 ± 0.90 cm, *p* = 0.001, respectively) and BFP (36.36 ± 1.50; 30.32 ± 0.43%, *p* = 0.001, respectively). In group 2, the tendency is inverse, i.e., women that underwent CT presented lower values for BMI, WC and BFP, but only for BFP was significantly lower (33.43 ± 1.66; 42.95 ± 1.03%; *p* = 0.000).

**Table 4:** Post hoc comparison for the chemotherapy factor between the different groups of tamoxifen use duration

Variables	Groups	Mean ± SD		*p-value	95% Wald Confidence Interval			
		Without CT	With CT		Without CT		With CT	
					Lower	Upper	Lower	Upper
BMI	1	25.29^1^ ± 0.46	29.14 ± 0.93	0.003	24.41	26.20	27.39	31.02
	2	28.40^1^ ± 0.95	26.76 ± 0.85	0.720	26.60	30.33	25.14	28.48
	3	21.97 ± 3.18	26.27 ± 0.83	0.720	16.55	29.18	24.70	27.94
WC	1	85.84^2^ ± 0.90	94.45 ± 1.96	0.001	84.10	87.62	90.68	98.38
	2	97.75^2,4^ ± 0.88	91.07 ± 2.44	0.105	96.03	99.50	86.41	95.97
	3	76.00^4^ ± 7.02	88.46 ± 2.07	0.429	63.40	91.10	84.49	92.61
BFP	1	30.32^3^ ± 0.43	36.36 ± 1.50	0.001	29.49	31.18	33.54	39.41
	2	42.95^3^ ± 1.03	33.43 ± 1.66	0.000	40.98	45.00	30.34	36.84
	3	36.30 ± 5.73	35.47 ± 1.65	0.988	26.64	49.47	32.39	38.85

^1^ *p* = 0.042; ^2^ *p* = 0.000; ^3^ *p* = 0.000; ^4^ *p* = 0.025; BMI, Body Mass Index; WC, waist circumference; BFP, body fat percentage; CT, chemotherapy; SD, Standard Deviation; Group 1, women using tamoxifen for the first 3 years; Group 2, women using tamoxifen between 3 and 4 years; Group 3, women using TMX for more than 4 years. Data adjusted for age, smoking, alcohol consumption, physical activity, energy (kcal), and clinical stage. *p calculated by ANOVA. Post hoc comparison (Sidak method)

Comparing women who underwent CT, no statistically significant differences were observed between the groups, even though mean values were higher in group 1 when compared to group 2 for BMI (29.14 ± 0.93; 26.76 ± 0.85, kg/m^2^, respectively), WC (94.45 ± 1.96; 91.07 ± 2.44 cm, respectively) and BFP (36.36 ± 1.50; 33.43 ± 1.66%, respectively) (Table 4).

Comparing women who did not undergo CT, we had significant differences between groups. Comparing women from groups 1 and 2, mean values were significantly lower for group 1 compared to group 2 for BMI (25.29 ± 0.46; 28.40 ± 0.95 kg/m^2^, *p* = 0.042, respectively), WC (85.84 ± 0.90; 97.75 ± 0.88 cm, *p* = 0.000, respectively) and BFP (30.32 ± 0.43; 42.95 ± 1.03, *p* = 0.000, respectively). Furthermore, comparing group 2 with group 3, for those women who did not undergo CT, mean values were lower for BMI, WC, and BFP, but only for WC the difference was significant (97.75 ± 0.88; 76.00 ± 7.02 cm, respectively, *p* = 0.025) (Table 4).

**Fig. 2:**
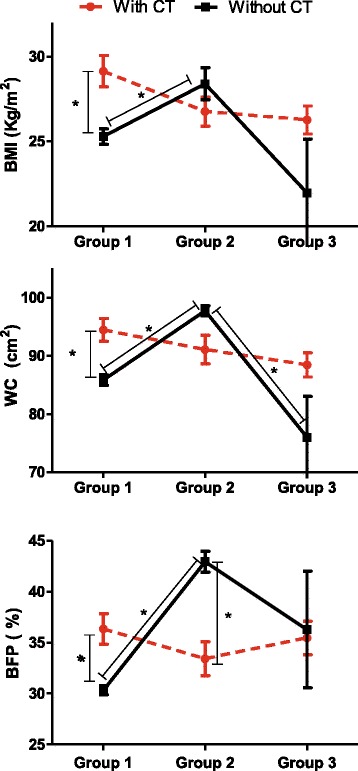
Distribution of women using endocrine therapy with TMX categorized according to groups (1, 2 and 3) and according to whether or not CT was performed. Distribution of women with breast cancer, according to groups of TMX usage duration in a university hospital in the city of Uberlandia, Minas Gerais, Brazil, 2015–2016 (*n* = 84, BMI and WC; *n* = 74, BFP). Group 1, women using tamoxifen for the first 3 years; group 2, women using tamoxifen between 3 and 4 years; Group 3, women using tamoxifen for more than 4 years; BMI, Body Mass Index; WC, waist circumference; BFP, body fat percentage; CT, chemotherapy; *p < 0.05 calculated by ANOVA. Post hoc comparison (Sidak method)

Figure 2 shows the post hoc comparisons for BMI, WC and BFP values of women who underwent CT and who did not undergo CT, grouped by TMX time usage (groups 1, 2 and 3).

## Discussion

In our study, we observed that the majority of women in endocrine therapy with TMX were classified as overweighed and obese, and we investigated the association of CT, usage time of TMX, and three different body parameters (BMI, WC and BFP). Although we did not find an isolated effect of CT, the interaction of CT with duration of TMX use showed a significant effect on BMI, WC and BFP. In our study, women from group 1 who did not undergo CT, presented lower values of body variables compared to those women who also did not undergo CT but were using TMX between 3 to 4 years (group 2). On the other hand, women from group 1 who underwent CT, presented higher values of body variables compared to those women who also underwent CT but were using TMX between 3 to 4 years (group 2). So, our study provides relevant knowledge to understand the need for specific and targeted conducts at different times of endocrine therapy.

In the present study we found values above the recommendations of weight and body fat excess in women on endocrine therapy with TMX, results similar to those observed in the literature [40, 41]. These body modifications related to increased adipose tissue lead to unsatisfactory outcomes, especially in postmenopausal women with BC [42–45]. However, these outcomes of weight gain during endocrine treatment with TMX are still controversial and need to be further investigated [13, 46, 47]. One of those outcomes could be an abnormally high expression of the aromatase enzyme in the breast, an enzyme that is responsible for the production of increased local estrogen, thus predisposing the mammary tissue to hyperplasia and cancer [18], as well as a bioenergetic adaptation of the cancer cells [48, 49].

In this sense, due to the important association of overweight with the prognosis of the disease [7–9, 27, 28], it is necessary to identify possible predictors about the body changes that occur during endocrine therapy with TMX. In the present study, we found that CT alone showed no effect on nutritional risk and obesity. It is known, however, that adjuvant CT for BC acts as an independent prognostic factor for bodily modifications with a potential long-term effect and may therefore affect the period of endocrine therapy [12, 13]. However, when we evaluated in this study the interaction between CT and duration of TMX use, we verified a significant effect on all body parameters evaluated, which demonstrates the relevance of that interaction in body changes over the years of endocrine treatment in the face of the increase of the number of long-term BC survivors and the many years of established endocrine therapy [3].

In this study, considering only women from group 1 (using TMX for the first 3 years), those that previously underwent CT had higher values of body fat and were more obese than those who did not undergone CT. The effect of CT interaction at different times of endocrine therapy with TMX on body parameters had not been reported in the literature before. Considering that the use of TMX starts in most cases after CT, the worst results for women who underwent CT may be due to the prolonged effects of chemotherapy and not for the effect of TMX. In a prospective and observational study performed with 272 French women treated with CT, greater weight changes were reported at 6 and 12 months after the end of this treatment [50], and the average weight gain in the first year after the end of the CT was 3 kg [51]. Such body modifications may be explained in part by the induction of CT in the reduction of energy expenditure [52], changes and perceptions of food due to the effects of nausea and changes in palatability [53], and negative nitrogen balance [54]. In addition, we may consider that the side effect of endocrine therapy with TMX on body weight, although still controversial, may exert an influence in this process. However, it is difficult to relate body modification entirely to TMX, since most studies of weight gain reports did not have a comparison group [13, 55, 56].

Also, an important aspect of CT is the induction of ovarian failure by treatment toxicity, especially in women approaching menopause [57, 58], and in Brazil the mean age of menopause is 51 years old [59]. A study with women with BC in CT found an immediate reduction of ovarian blood flow after treatment, demonstrating a postmenopausal profile for most patients accompanied by related symptoms [60]. Thus, those perimenopause women who do CT, especially with anthracycline-based regimens compared to CMF [61], may enter menopause more frequently with CT and present earlier and induced symptoms already known from climacteric, such as changes in body composition [62, 63].

When analyzing women who did not have CT in this study, we verified that the highest nutritional risk and body fat did not occur in the group with at most 3 years of TMX use, but in women in the intermediate duration group, between 3 and 4 years. Results of a cross-sectional study with american women found that the highest percentage of weight gain occurred after 3 years of TMX use; however, CT was not considered [4]. These results suggest that these women who do not have CT may have different reactions between them. First, the concentration of important metabolites of TMX oxidative metabolism, such as endoxifene, is related to the occurrence of side effects from drug use [4], suggesting the need for prospective studies to see if different concentrations occur throughout treatment and its relation to previous treatments, such as CT.

Also, food intake is an important modifiable factor contributing to changes in nutritional status and the risk of obesity in women with BC [52–54]. However, in our study, we found that women did not present statistically significant differences for the average amount of energy, carbohydrate and protein intake among the three groups evaluated. However, only significantly higher mean values ​​for lipids were observed for women in group 1. Possibly, the preference for more palatable foods in this period, still resulting from the cytotoxicity of those who did CT [53], may have influenced this result. In addition, as a result of the several years proposed for endocrine therapy [3], it has been shown that psychological factors such as anxiety and depression are common in endocrine therapy [64], and may interfere with changes in the dietary pattern [65]. There is a need for prospective studies to evaluate and consider these factors for better explaining these findings in view of the negative effects of obesity.

Additionally, we found altered values for TG and HDL-C, with HDL-C showing inadequate values in women in the first 3 years and between 3 and 4 years of treatment statistically significant between the three groups. In this sense, since central obesity is associated with several biochemical alterations, including decreased glucose tolerance, elevated serum insulin levels and lipid changes [41–43], blood assessments are important in this population, being a risk factor for many diseases associated with such changes, including diabetes mellitus and cardiovascular disease [66, 67].

In general, adjuvant therapy with AI is associated with better outcomes compared to TMX for postmenopausal women with endocrine-responsive BC. However, in many public hospitals from Brazil (as in our case), taken into consideration cost issues, AIs are reserved to be used only in high risk early BC patients. This approach is not all bad, considering findings from the Breast International Group Trial 1–98 comparing adjuvant TMX with letrozole which showed considerable less benefit of AI over TMX in patients presenting lower risk of recurrence [68, 69]. However, many postmenopausal women with endocrine-responsive BC are still receiving TMX in low-resource hospitals. In addition, TMX has been chosen for patients presenting moderate to severe osteoporosis (which is not uncommon in post-menopausal women). Also, it is important to mention that central obesity becomes more prevalent after menopause, which may have distorted the results.

Possible limitations of this study should be considered. One limitation is the use of unequally weighted populations relative to the menopausal state that may at least in part interfere in the generalization of the study. Moreover, cross-sectional evaluation makes it impossible to establish causal relationships with changes in body composition and duration of TMX use along with the other variables. In this way, it would be important to obtain the usual weight before the beginning of the treatments for BC, since that obesity may have had some correlation with BC incidence and some effect on the treatment regimen chosen in the first place, and that is a possible confounder when interpreting the data from this study. We did not evaluate the CT in relation to its different chemotherapeutic agents, in which they can respond differently to weight gain [70].

## Conclusions

Our results suggest that women in endocrine therapy with TMX require nutritional monitoring throughout treatment with the need for targeted interventions at specific times. Women who have undergone CT prior to initiating endocrine therapy deserve special attention in the first 3 years of treatment. However, women who did not undergo CT had a higher nutritional risk in the intermediate treatment period (between 3 and 4 years). In view of the great benefit of endocrine therapy with TMX already established, which exceeds the negative effects on body composition, these results reinforce the importance of nutritional guidelines and multidisciplinary follow-up, taking into account previous treatments such as CT, thus ensuring that BMI and body composition are reduced or maintained within a healthy range. In addition, these strategies may contribute to a greater adherence to treatment and also better medication action.

## Abbreviations

24HR: 24-h dietary recall
AC: Adriamycin + Cyclophosphamide
BC: Breast cancer
BFP: Body fat percentage
BIA: Bioelectrical impedance
BMI: Body mass index
CMF: Cyclophosphamide, methotrexate, and 5-fluorouracil
CRP: C-Reactive protein
CT: Chemotherapy
FAC: Cyclophosphamide, doxorubicin, and 5-fluorouracil
G1: Well-differentiated tumor (low grade)
G2: Moderately differentiated tumor (intermediate grade)
G3: Poorly differentiated tumor (high grade)
GEE: Generalized estimating equations
Group 1: Women using tamoxifen for the first 3 years
Group 2: Women using tamoxifen between 3 and 4 years
Group 3: Women using TMX for more than 4 years
HDL-C: High density lipoprotein
LDL-C: Low density lipoprotein
NR: Not reported
TG: Triglycerides
TMX: Tamoxifen
WBC: White blood cell count
WC: Waist circumference
